# Disambiguate: An open-source application for disambiguating two species in next generation sequencing data from grafted samples

**DOI:** 10.12688/f1000research.10082.2

**Published:** 2017-01-24

**Authors:** Miika J. Ahdesmäki, Simon R. Gray, Justin H. Johnson, Zhongwu Lai

**Affiliations:** 1 AstraZeneca IMED Oncology, Cambridge, UK; 2 AstraZeneca R&D Information, Cambridge, UK; 3 AstraZeneca Oncology iMed, Waltham, USA

**Keywords:** NGS, patient derived xenograft, explant, disambiguation, sequencing

## Abstract

Grafting of cell lines and primary tumours is a crucial step in the drug development process between cell line studies and clinical trials. *Disambiguate *is a program for computationally separating the sequencing reads of two species derived from grafted samples. *Disambiguate *operates on DNA or RNA-seq alignments to the two species and separates the components at very high sensitivity and specificity as illustrated in artificially mixed human-mouse samples. This allows for maximum recovery of data from target tumours for more accurate variant calling and gene expression quantification. Given that no general use open source algorithm accessible to the bioinformatics community exists for the purposes of separating the two species data, the proposed *Disambiguate *tool presents a novel approach and improvement to performing sequence analysis of grafted samples. Both Python and C++ implementations are available and they are integrated into several open and closed source pipelines. *Disambiguate *is open source and is freely available at https://github.com/AstraZeneca-NGS/disambiguate.

## Introduction

Xenografts, both cell line and primary tumour, are routinely profiled in preclinical and translational research. Xenografts are used to study everything from new target identification to responses to targeted therapeutics and mechanisms of resistance ^1^ in an environment that is more realistic than just 2D cell lines. However, due to mouse stromal contamination of the human tumour, not all the data resulting from studying the extracted samples are guaranteed to be of human origin.

Direct high throughput sequencing of grafted samples with a mixture of two species is routine practice. However, the origin species of each read or read pair is unknown and needs to be determined informatically. With the high volume of data and computational challenges of alignment and kmer identification, new computational strategies are required to computationally separate the two species’ components for more accurate downstream analysis ^1^, especially for the reduction of variant calling artefacts. However, the two-species alignment approach proposed in Bradford *et al*. ^1^ excludes reads that align to both organisms, clearly dismissing a large portion of the data as evidenced in Table 1 when observing cross species alignment rates.

Algorithms designed for disambiguating the host and tumour sequences include e.g. the xenome tool ^2^, which is based on analysing k-mers from both species and performing simple set operations to assign reads to either species. Xenome was made available as open source via the gossamer repository after the initial publication of this manuscript and therefore results from xenome are now included in an updated comparison. In 3 the authors also aligned the reads to both species, but no attempt was taken to disambiguate the data and no implementation is readily available.

**Table 1.** Read pairs assigned human (hg19) and mouse (mm10) by both the disambiguate and xenome algorithms. The ’ambiguous’ column includes reads that aligned but could not be unambiguously disambiguated. The ^†^ symbol and the numbers in parentheses indicate false positive reads prior to applying the disambiguation algorithm on the raw alignments. TP denotes true positive and FP false positive.

Tool	Material	Sample	Total reads	Mouse mm10	Human hg19	ambiguous
disambiguate	DNA	SRR1176814 (mouse)	47312349	47197650 (99.76%) TP	26157(0.06%) FP (25638785 (54.19%)) ^†^	88542 (0.19%)
xenome	DNA	SRR1176814 (mouse)	47312349	46889894 (99.11%) TP	20031 (0.04%) FP	339326 (0.72%)
disambiguate	DNA	SRR1528269 (human)	77268164	11502 (0.01%) FP (39686392 (51.36%)) ^†^	77102895 (99.79%) TP	153767 (0.20%)
xenome	DNA	SRR1528269 (human)	77268164	3291 (0.004%) FP	76593625 (99.13%) TP	521239 (0.67%)
disambiguate	RNA	SRR1930152 (mouse)	24056144	23126086 (96.13%) TP	80694 (0.34%) FP (3005372 (12.49%)) ^†^	849364 (3.53%)
xenome	RNA	SRR1930152 (mouse)	24056144	23071432 (95.91%) TP	43294 (0.18%) FP	625302 (2.60%)
disambiguate	RNA	SRR387400 (human)	59653070	94289 (0.16%) FP (6001230 (10.06%)) ^†^	49677937 (83.28%) TP	9880844 (16.56%)
xenome	RNA	SRR387400 (human)	59653070	83621 (0.14%) FP	53851984 (90.28%) TP	2043780 (3.43%)

Here, an alternative approach using read alignment quality is proposed to further disambiguate reads that can be mapped to both species. Alignment is first performed to both species independently and the reads are disambiguated as a post-processing step, assigning reads to the species with higher quality alignments. There is no requirement to maintain pseudo reference indices based on combinations of reference sequences. This approach shows a very high sensitivity and specificity on artificially generated samples obtained by mixing reads from the individual species. The *Disambiguate* tool is community supported and widely used in several open and closed source pipelines.

## Methods

### Implementation

**Figure 1.**
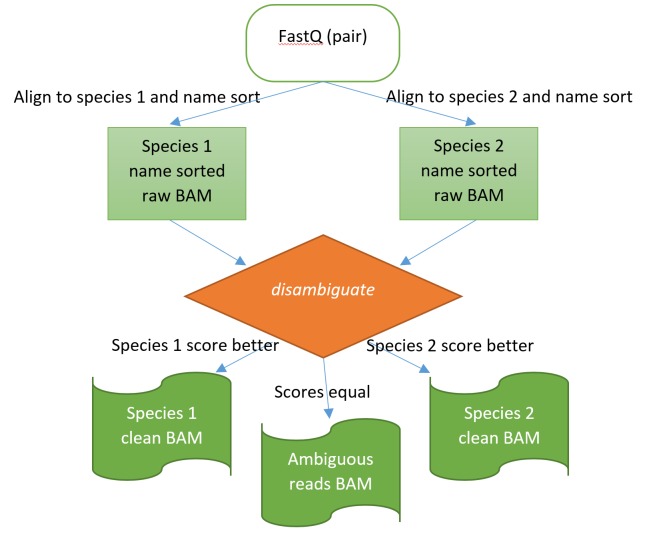
The disambiguation process illustrated. Alignment is first performed against both species. The disambiguation application then operates on the raw, natural name sorted BAM files to assign the read pairs into one of the two species or as ambiguous for unresolved cases.

The *Disambiguate* algorithm works by operating on natural name sorted BAM files from alignments to two species. Name sorting is a critical part in not having to read all the data from both species’ alignments into memory simultaneously; the same read aligned to both species is disambiguated on the fly by going through both alignment files synchronously. For reads that have alignments to both species and therefore require disambiguation, the specific details of the disambiguation process are slightly different for the different aligners. Thus far the algorithm has been tested for BWA MEM ^4^ and Bowtie2 ^5^ for DNA-seq, and TopHat2 ^6^, STAR ^7^ and Hisat2 ^8^ for RNA-seq. Illumina’s paired end sequencing is preferred as the mate can often break a tie. Figure 1 illustrates the disambiguation process.

*Disambiguate* assigns the reads on a per-pair basis, based on the highest quality alignment of the read pair. For BWA and STAR the alignment score (AS tag, higher better) is used as the primary disambiguation metric followed by edit distance (NM, lower better) to the reference for any ties.

Allowing multiple alignments, let *QS* be an array of size 4 of the highest quality scores (AS primarily, –NM secondarily) for all read 1 species 1, read 2 species 1, read 1 species 2 and read 2 species 2. Then

1. If max(QS _1,2_) > max(QS _3,4_) or max(QS _1,2_) == max(QS _3,4_) and min(QS _1,2_) > min(QS _3,4_) assign to species 1
2. If max(QS _1,2_) < max(QS _3,4_) or max(QS _1,2_) == max(QS _3,4_) and min(QS _1,2_) < min(QS _3,4_) assign to species 2
3. If AS did not resolve, repeat for –NM
4. If neither AS nor –NM resolved, assign ambiguous

For Tophat2 and Hisat2 based alignments the sum (lower better) of edit distance, number of reported alignments (NH) and the number of gap opens (XO) is used. Let *QS* = *NM* + *NH* + *XO*. Then

1. If the scores are identical for the highest ranking reads for both species, assign ambiguous
2. If min(QS _1,2_) < min(QS _3,4_) or min(QS _1,2_) == min(QS _3,4_) and max(QS _1,2_) < max(QS _3,4_) assign to species 1
3. Else assign to species 2

Aligner tags for BWA and STAR are almost identical, as are the aligner tags for Tophat2 and Hisat2. However STAR and BWA lack most of the tags used by Tophat2/Hisat2, for which the original disambiguation scheme was developed. This is the underlying reason for using two separate schemes. Relative weighting schemes could potentially also be considered for the tag values to improve sensitivity and specificity. This would run the risk of overfitting to the data though and would need to be evaluated over a very large data set.

### Operation

The algorithm is implemented in Python (with dependency on the Pysam package) and C++ (with dependency on BamTools), with the C++ version being approximately four times faster than the Python code. 64 bit unix/linux systems are supported.

Given name sorted alignment (BAM) files aligned to the two species of interest (e.g. human and mouse), the algorithm infers for each read the most likely origin. The output contains BAM files for both species, BAM files for ambiguous reads and a text file describing how many read pairs were assigned to each BAM file. The simplest way to perform all of the alignment and disambiguation is by running bcbio, in which *Disambiguate* is integrated, on the raw sequencing data.

## Results

To illustrate the utility of *Disambiguate*, raw publicly available human and mouse sequencing data was downloaded. First exome sequencing reads (100bp paired end Illumina data) were obtained from the European Nucleotide Archive ( ENA) with Run Accessions SRR1176814 and SRR1528269.

The reads were aligned against hg19 and mm10 using BWA MEM, and processed using *Disambiguate* and xenome. Pre-disambiguation, for the human sample (SRR1528269), there were 39686392 read pairs (out of total 77268164), for which at least one read aligned to mouse. Similarly, for the mouse sample (SRR1176814), there were 25638785 read pairs (out of total 47312349) for which at least one read aligned to human. Table 1 summarises the disambiguation results. As can be seen, the disambiguation algorithm correctly pulls apart virtually all of the read pairs. *Disambiguate* shows slightly more true and false positives in comparison to xenome. In other internal studies, *Disambiguate* has time and again highlighted samples with low human assigned component, correlating with poor extraction or lack of growth of the tumour cells in the host.

For RNA-seq, STAR aligned human (SRR387400) and mouse (SRR1930152) data was also analysed with very similar results. For the mouse sample *Disambiguate* displays again slightly more true and false positives compared to xenome but for SRR387400 xenome shows clearly more true positives.

## Conclusions

In summary, *Disambiguate* provides an important tool for computationally separating sequence reads originating from two species. In human-mouse studies it also allows the study of the mouse stromal component for gene expression and DNA variation. The results presented here show excellent separation of the host and graft. Future work includes evaluating how the performance is affected by the use of very highly mutated tumour xenografts based on for example MCF7.

In addition to RNA-seq and whole genome sequencing, it is worth highlighting that for targeted hybridisation capture sequencing of xenograft samples, where baits from a single species are used, disambiguation is still highly recommended. This is best seen in Table 1 where a large number of human exome reads aligned to mouse and would potentially affect downstream interpretation without disambiguation.

*Disambiguate* has been well adopted in the open source community; it is integrated in the open source bcbio pipeline and has been successfully used in both RNA and DNA sequencing of xenografts both at AstraZeneca and other research institutes. This is evidenced by the number support tickets from a variety of organisations on the bcbio-nextgen Github page.

## Data availability

The data referenced by this article are under copyright with the following copyright statement: Copyright: © 2017 Ahdesmäki MJ et al.

Data associated with the article are available under the terms of the Creative Commons Zero "No rights reserved" data waiver (CC0 1.0 Public domain dedication). 

The data used here is available from the European Nucleotide Archive with Run Accession numbers SRR1176814 and SRR1528269.

## Software availability

Software integrating *Disambiguate* available from: https://github.com/chapmanb/bcbio-nextgen

Latest source code: https://github.com/AstraZeneca-NGS/disambiguate

Archived source code as at time of publication: DOI: 10.5281/zenodo.166017

License: MIT
