# Supplementary material for: Disambiguate: An open-source application for disambiguating two species in next generation sequencing data from grafted samples
Source: F1000Res. 2017 Jan 24;5:2741. Originally published 2016 Nov 22. [Version 2] doi: 10.12688/f1000research.10082.2 (PMC5130069; doi:10.12688/f1000research.10082.2)
Supplement: Peer review history

# Peer review: Referee response for version 1

**DOI:** 10.5256/f1000research.10863.r17881

**Published:** 2016-12-05

**Authors:** Gavin R. Oliver, Asha Nair

**Affiliations:** 1 Department of Health Sciences Research, Mayo Clinic, Rochester, MN, USA; 2 Mayo Clinic

We believe that overall the software tool article by Ahdesmäki *et al. *seems sound and provides a solution to a problem that appears to be inadequately addressed in the field currently. 

 Nonetheless, we believe the manuscript would benefit from some minor amendments in order to increase its utility and accessibility to readers.

 In brief:

**Intro/Background**

 Needs expanded slightly to better set the scene and describe the general approach of read disambiguation.

**Methodology**

 The methodology should be expanded slightly and made more explicit.

**Tables 1&2:**

- Combine 1 & 2 into a single table and label the samples by data type, i.e DNA and RNA
- Show %s as well as numbers
- Clearly label the species in the tables
- Clearly label correctly mapped/incorrectly mapped reads in table
- Clearly label human and mouse genomes as such
- Tables should clearly show all numbers pre- and post- disambiguation, rather than having superscripted references in the table legend
- Essentially, a novice should be able to read the paper and extract relevant info more easily.

**Figure 1 **

- Should be more granular, informative and descriptive of the process. Include read alignment etc.  Describe the Disambiguate process
- Use same font size for all text in the Figure

**Comparison with a competitor product**

 This is something that is clearly missing. If it is literally impossible to compare to a competitor because the software is not accessible, this should be stated clearly as a reason for the lack of comparison in the paper.

**Tumor samples**

 It would be interesting to know how performance is affected by use of highly mutated tumor xenografts. This is arguably beyond the scope of the paper, but warrants at least some mention.

We have read this submission. We believe that we have an appropriate level of expertise to confirm that it is of an acceptable scientific standard, however we have significant reservations, as outlined above.

# Response

**Published:** 2017-01-11

**Authors:** Miika Ahdesmäki

Dear Gavin and Asha, 

 Many thanks for the very detailed review and comments. We have addressed your points in v2 of the manuscript.  

 Into/background: 

 We have added the text in braces: "Direct high throughput sequencing of grafted samples with a mixture of two species is routine practice. {However, the origin species of each read or read pair is unknown and needs to be determined informatically.}" to better set the scene. Further, the operation of xenome is now updated and xenome is now included in a comparison study. We have more explicitly stated that "Alignment is first performed to both species independently and the reads are disambiguated as a post-processing step, {assigning reads to the species with higher quality alignments}" 

 Methodology: 

 We have clarified the methodology section by spelling out the disambiguation algorithm and giving the reasoning why two schemes are used.  

 Table 1&2: 

 We have combined Tables 1&2 and revised the contents to address these points. 

 Figure 1: 

 We have redrawn the figure to be more descriptive. 

 Comparison to competitor product: 

 We have now compared our approach to Xenome, which was recently open sourced, and included the results of the comparison in the updated table with discussion. 

 Tumor samples: 

 We agree that evaluating the performance of the disambiguation algorithm in a messy cancer genome like the highly rearranged MCF7 would be extremely interesting. If we get our hands on appropriate data we will consider publishing the results on the program Github page.

# Peer review: Referee response for version 1

**DOI:** 10.5256/f1000research.10863.r17879

**Published:** 2016-11-25

**Authors:** Matthew D. Eldridge

**Affiliations:** 1 Cancer Research UK Cambridge Institute, University of Cambridge, Cambridge, UK

This paper describes a computational tool for separating sequencing reads from a sample that contains DNA or RNA from two species. This is a necessary pre-processing step for genomic or transcriptomic analysis of patient-derived xenograft cancer models.

 The approach is based on alignments of sequence reads to the reference genome sequences for the two species in question. The authors have tested their approach on DNA-seq data from publicly available human and mouse exome datasets concatenated to simulate a xenograft sample. The results presented in Table 1 show very good separation of reads from the two species datasets with only a small percentage of reads being assigned to the wrong species (0.06% and 0.01%) and a higher but still very low percentage of reads flagged as ambiguous, i.e. align equally well to both genomes. Similar results were presented for RNA-seq data, although here the percentages of incorrectly assigned and ambiguous reads are unsurprisingly higher than for DNA-seq.

 Use of the alignment scores, and in the event of a tie the edit distance, is a reasonable approach to disambiguate reads and is the method used for BWA and STAR alignments. For TopHat2 and HISAT2 a different scoring function is required, although the reasons for this are not given. Further, the choice of function (sum of edit distance, number of reported alignments and number of gap opens) is not completely obvious and raises the question of whether the authors have attempted to tune the function, e.g. by adjusting the weighting of each component.

I have read this submission. I believe that I have an appropriate level of expertise to confirm that it is of an acceptable scientific standard.

# Response

**Published:** 2017-01-11

**Authors:** Miika Ahdesmäki

Dear Mathew, 

 Many thanks for reviewing our manuscript and the comments. We have modified v2 of the manuscript to address the points you raise, namely: 

1. The aligner tags are very similar between BWA and STAR; and between TopHat2 and HISAT2. However, fairly different between BWA/STAR vs TopHat2/Hisat2 and therefore we couldn't use the same scheme originally developed for TopHat2 with BWA/STAR. With the appearance of HISAT2 especially for hg38 we decided to utilise the TopHat2 scheme for HISAT2 given their outputs are almost interchangeable. We have mentioned this in the updated text. 
2. The sum of edit distance, number of reported alignments and number of hap opens has always worked for us well out of the box (as illustrated in the tables) and while tuning their weights may yield some minor benefits, it would risk overfitting to existing data. Any benefits of the weight tuning would have to be measured over a very long time, running multiple versions of weighted and the unweighted algorithms side by side. We have given this reasoning (complexity) in the text as our excuse of not tuning the weights further.  

 

 Thank you again for the comments and helping us improve the manuscript.

# Peer review: Referee response for version 1

**DOI:** 10.5256/f1000research.10863.r17877

**Published:** 2016-11-23

**Authors:** Daniel Nicorici

**Affiliations:** 1 Orion Corporation Orion Pharma, Espoo, Finland

This papers introduces a tool, named Disambiguate, for computationally separating the DNA/RNA sequencing reads of two species, like for example in case of xenograft samples. The tool takes as input BAM files from wide range of NGS aligners.

 I have made the following minor observations: 

1. The tool Disambiguate works on RNA-seq and DNA-seq data and this is mentioned for the first time in Methods section. Probably it would help to have this mentioned much earlier, like for example in the abstract too.
2. In order to improve the clarity, to the Tables 1 and 2 could be added also the percentages where is relevant, like for example, "26157" would become "26157 (0.0553%)" and so on.

I have read this submission. I believe that I have an appropriate level of expertise to confirm that it is of an acceptable scientific standard.

# Response

**Published:** 2017-01-11

**Authors:** Miika Ahdesmäki

Dear Daniel, 

 Thank you for the review, your comments are much appreciated. We have addressed your points in v2 of the manuscript. 

1. We have explicitly mentioned in the abstract and the introduction that the tool can be used for both DNA and RNA-seq data 
2. We have added percentages into the tables as you suggested 

Thank you for the review and helping us improve the manuscript.
